# Lessons Learnt from Evidence-Based Approach of Using Chinese Herbal Medicines in Liver Cancer

**DOI:** 10.1155/2013/656351

**Published:** 2013-05-29

**Authors:** Zhan Zheng, William Chi-Shing Cho, Ling Xu, Juyong Wang, Daniel Man-Yuen Sze

**Affiliations:** ^1^Department of Oncology, Longhua Hospital, Shanghai University of Traditional Chinese Medicine, Shanghai 200032, China; ^2^Department of Clinical Oncology, Queen Elizabeth Hospital, Kowloon, Hong Kong; ^3^Department of Health Technology and Informatics, The Hong Kong Polytechnic University, Kowloon, Hong Kong

## Abstract

This paper is a systematic review of evidence-based studies of the effectiveness of Chinese herbal medicine (CHM) in the treatment of liver cancer. After a detailed analysis of the literature, five animal studies and four human clinical trials met the criteria for inclusion. Analysis revealed that results of the clinical trials, whilst encouraging, need to be interpreted with caution as problems with study designs may lead to apparent benefits being attributable to various forms of bias. However, as each of the CHM agents used in these studies appeared to be potentially beneficial, further well-designed and controlled randomized clinical trials are warranted. The second part of this review focused on the lessons learned from the relationships between Traditional Chinese Medicine (TCM) theory, TCM Syndrome Differentiation, and modern scientific understanding of mechanisms of action of CHM agents. The understanding of TCM Syndrome Differentiation may allow identification of different patterns of disharmony and may provide important guidance to the prescription of CHM. Furthermore, quality control using both biological and chemical fingerprinting of CHM is important to ensure batch-to-batch consistency to deliver sustained therapeutic benefit. Also, careful assessment of herb-drug interactions is paramount for safety and integrative use of western chemotherapeutic and CHM agents.

## 1. Introduction

This review aimed to examine the evidence for using Chinese herbal medicine (CHM) in cancer treatment in terms of the benefits and potential mechanisms of action. As the same CHM formulae may be used for different cancer types with different effectiveness, in this review, liver cancer or hepatocellular carcinoma (HCC) has been used as the specific focus for discussion. Liver cancer is the second leading cause of cancer death in men and the sixth in women worldwide with an estimated 748,300 new liver cancer cases and 695,900 liver cancer deaths occurring in 2008 [1]. The highest liver cancer rates are found in East and South-East Asia and in Middle and Western Africa. In the United States between 1990 and 2007, the increase in death rates for liver cancer accounted for nearly 70% of the total increase in the cancer death rates in men and for almost 23% of the increase in women [2]. 

Liver cancer has a poor prognosis, and the 5-year survival rate reported between 1973 and 2007 remained below 12% in the United States [3]. Early stage liver cancer is currently difficult to diagnose due to the lack of a sensitive screening test. As a result, only 30% to 40% of patients with liver cancer are candidates for potentially curative treatments at the time of their diagnosis [4]. Treatment modalities such as surgical resection, liver transplantation, and local ablation are only considered for patients with preserved liver function. However, most newly diagnosed liver cancer patients are already at an advanced stage. For these intermediate or late stage liver cancer patients, the therapeutic options are limited to palliative approaches using transcatheter arterial chemoembolization (TACE) or chemotherapeutic agents [3]. However, many patients are either not suitable for TACE or suffer from poor outcomes with conventional systemic cytotoxic chemotherapy [5]. 

A meta-analysis summarized the results of 193 randomized controlled trials (RCTs) of a range of medical modalities for HCC treatment reported during 2005–2010 indexed by MEDLINE, CANCERLIT, Embase databases, and the Cochrane Library [6]. From the 32 studies that met their inclusion criteria, only 17 studies were eventually selected based on the strength of the trials. Of these, one study used chemoembolization, three used tamoxifen hormonal treatment, and a further three employed systemic chemotherapy treatment modalities such as doxorubicin and PIAF—cisplatin/interferon alpha 2b/doxorubicin/fluorouracil. The researchers summarized the effects of all these treatment modalities for HCC in the category of “No survival benefit.”

New conventional medicine treatment approaches for HCC now rely on molecular targeted therapies such as the multikinase inhibitor Sorafenib. In a recent randomized multicenter placebo-controlled phase III trial, Sorafenib exhibited a benefit in advanced liver cancer patients by extending overall survival, but this was only by 2 to 3 months compared with the placebo [7]. Therefore in view of the poor clinical effectiveness of a broad range of medical modalities including the molecular targeted approach, new therapeutic agents with low cost and high effectiveness, such as herbal medicines, are urgently needed.

Natural products represent a rich reservoir of potential bioactive compounds exhibiting anticancer properties [8]. Several compounds have been found to have powerful antitumor effects, such as taxol, which was identified by screening approximately 114,000 plant extracts and obtained therapeutic approval from the Food and Drug Administration of the United States [9]. Although herbal medicines have been used in clinical applications for centuries, their present use lacks stringent supporting scientific evidence in terms of double-blind placebo-controlled clinical trials. In addition, since there are usually multiple compounds in a single herb, the actual active compounds and their sites of action and mechanisms of action are generally unknown. The situation is further complicated by the use of herbal medicines as a formula with multiple components [10–12], which represents a “polychemical” approach in contrast to the “single-chemical” approach of classical chemotherapeutics.

CHM has been practiced for centuries by local physicians caring for a huge population in China and in East Asia including Korea and Japan and has developed a comprehensive set of well-documented medical theories. CHM usually requires the use of multiple herbs, minerals, or even compounds derived from animal parts. In light of CHM theory, management of health can be characterized as holistic with the emphasis on regulating the integrity of the human body functions and the interaction between various organs and the internal environment. Likewise, liver cancer is a systemic disease associated with a local tumor. Therefore, CHM therapies not only focus on eliminating the local malignancy, but also aim to restore the homeostasis of the whole body.

This review focuses on the current understanding of CHM as a therapy for liver cancer and explores approaches for its future development as an evidence-based complementary and alternative medicine (CAM) for liver cancer management. Based on the premise that a successful CHM cancer treatment would require the clinical efficacy substantiated by clinical trials in humans, this review aims to first present a critical analysis of the clinical studies and the related preclinical animal *in vivo* studies, followed by experimental *in vitro* results which can help to delineate the underlying mechanisms of action of CHM treatment. This review highlights some important lessons from critical analysis of the CHM anti-HCC research, including inadequacies in the reported clinical trials, possible CHM candidates for future clinical trials, and quality control of CHM to ensure batch-to-batch consistency. These considerations may contribute to enhancing the development of an evidence-based cancer research platform using CHM.

## 2. Preclinical and Clinical Studies of CHM in Liver Cancer

### 2.1. Study Selection

An extensive search was performed in four electronic databases (PubMed, EMBASE (Excerpta Medica Database), China Biological Medicine database, and CNKI (Chinese Journal Full-Text Database for clinical trials and animal studies of CHM-based therapy) targeting liver cancers. The database search period covered 1980 to February 2012. The search terms were comprised of the following combinations: “Chinese herbal medicine,” “Chinese herb,” “herbal medicine,” “CHM,” “traditional Chinese medicine,” “TCM,” “liver cancer,” “hepatocellular carcinoma,” “cholangiocarcinoma,” and “hepatic carcinoma.” No other restrictions were imposed. In addition, the reference lists of recent reviews related to this topic were examined.

**Figure 1:**
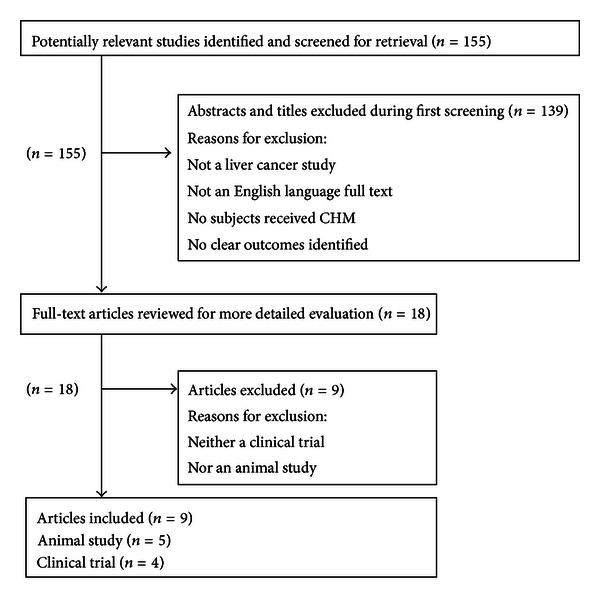
Process of study selection.

The flow chart demonstrating the selection process of the systematic analysis is shown in Figure 1. An initial screen of identified abstracts or titles was performed followed by a thorough reading of selected full-text articles. Studies were considered eligible if they met the following criteria: (1) the study design was a clinical trial or animal study; (2) the language of publication was English; (3) the main exposure of interest was CHM; (4) the outcome of interest was treatment effects for liver cancer. Using the previous criteria, 30 clinical trials in the area of integrative CHM with western medicine for treating liver cancer reported in Chinese were excluded from this review [13]. This review focused on the evaluation of four CHM formulas or extracts in human trials and five related preclinical animal experiments targeting liver cancer. We attempted to critically analyze the reports of use of these CHM extracts or formulas in clinical trials (216 liver cancer patients in total). A meta-analysis was not performed because of the small number of identified studies.

### 2.2. Preclinical Studies in Animal Models and Anticancer Mechanism of CHM

Effects of five CHM extracts of Bufalin, *Scutellaria barbate D. Don*, Kanglaite injection, PHY906, and Ganfujian or formulas on liver cancer have been reported in animal models (Table 1).

#### 2.2.1. Bufalin

Bufalin, a cardiotonic steroid isolated from the skin and parotid venom glands of Bufo toad (*Bufo bufo gargarizans Cantor*), is a major active component of Huachansu [20]. The molecular formula of bufalin is C_24_H_34_O_4_ with a relative molecular weight of 386.5 g/mol. To investigate the antitumor activity and apoptosis-regulating mechanism of bufalin in an animal model, human BEL-7402 tumors were implanted into the liver of 75 nude mice to establish orthotopic transplantation tumor models [14]. This simulation used a novel intrahepatic tunnel implantation to establish the *in situ* hepatoma. Compared to the murine tumor model, in which cancer cells are subcutaneously transplanted in immune-deficient animals, this *in situ* transplantation model is closer to the clinical situation of liver cancer. The results indicated that bufalin alone could inhibit growth, leading to tumors with a significantly smaller size (35.21 ± 12.51 cm^3^) compared with those in the normal saline (NS) (170.39 ± 25.29 cm^3^; *P* < 0.01) and Adriamycin (ADM) groups (55.17 ± 16.13 cm^3^; *P* < 0.05). Bufalin also prolonged survival times of hepatoma-bearing mice compared with those in the NS group and ADM groups (31.8 ± 4.2 d versus 23.4 ± 2.1 d, *P* < 0.05, and 31.8 ± 4.2 d versus 22.2 ± 1.6 d, *P* < 0.05, resp.).

In addition to these effects, apoptosis was induced by bufalin which regulated the expression of an apoptosis-related gene, Bax, and the ratio of Bcl-2/Bax. Apoptotic characteristics such as cell shrinkage, cytoplasm concentration, and apoptotic corpuscles were also determined by ultrastructure observation. These results suggested that bufalin treatment can alleviate the tumor burden and prolong survival in the mouse model. Clinical trials should be inaugurated to test the efficacy of bufalin in liver cancer patients.

#### 2.2.2. Kanglaite

Kanglaite is an acetone extract of Coix seed (*Semen Coicis*) that is widely used in clinical cancer therapy in China. CHM theory states that *Semen Coicis* increases the energy of the body and reduces or eliminates nodes, as well as benefiting digestive disorders [21].

Effects of Kanglaite injection were investigated using rat models of transplanted Walker-256 hepatoma [15]. Forty hepatoma-bearing Wistar rats were treated with intratumor injections of Kanglaite and compared with a control group receiving saline treatment. The hepatoma volumes of rats in the Kanglaite group were significantly smaller than those in NS group (235.4 mm^3^ versus 464.6 mm^3^, *P* < 0.05). The tumor inhibitory rate in the Kanglaite group was 49.4%, and the serum levels of alanine aminotransferase, related to hepatic function, were significantly lower. Inhibition of proliferating cell nuclear antigen (PCNA) expression indicated that one of the antitumor mechanisms of Kanglaite could be inhibition of karyokinesis and propagation of cancer cells. The results indicated that intratumor injection of Kanglaite could effectively inhibit hepatoma in rats. Kanglaite was also reported to induce *in vitro* apoptosis of HepG2 cells through the activation of the Fas/FasL pathway [16]. Although further studies of the mechanisms are needed, these findings have contributed to the understanding of Kanglaite's anticancer activity.

#### 2.2.3. *Scutellaria Barbate D. Don*

*Scutellaria barbate D. Don*, a perennial herb growing throughout southern China, is known as *Banzhilian* in CHM. According to CHM theory, it can be used to eliminate toxicity, promote blood circulation, and reduce tumor nodes. It has been widely used as a therapy for cancers of the liver, lung, stomach, and breast, as well as colorectal cancer [22]. In an *in vivo *study of hepatoma investigating the antitumor effect and the mechanisms of a crude extract of *Scutellaria barbate *(SB) [17], 60 ICR H22 hepatoma-bearing mice were treated with SB, fluorouracil (5-FU), and NS. SB significantly inhibited tumor growth compared to use of NS (1.67 ± 0.76 g, 2.65 ± 1.12 g, resp.; *P* < 0.05). In addition, SB significantly improved the phagocytotic function of macrophages, which was analyzed by comparing the chicken-red cell phagocytic rate, with both 5-FU and NS groups (*P* < 0.05). The phagocytotic function of macrophages can reasonably reflect the antitumor immune function. The study demonstrated the antitumor activity of SB in H22-bearing mice and suggested that a potential mechanism was the improvement of immune function. This study also demonstrated that SB was able to inhibit the proliferation of H22 cells *in vitro* in a dose- and time-dependent manner by use of MTT assays. Observation of ultrastructure revealed apoptosis of H22 cells induced by SB. Further detailed investigation of the mechanisms involved in the observed antitumor effects of SB is warranted.

#### 2.2.4. PHY906

PHY906 is derived from the formulation known as Huang Qin Tang, which was first described in CHM documents dating approximately 1,800 years ago and is used for the treatment of various gastrointestinal symptoms, including diarrhea, nausea, and vomiting [23]. PHY906 consists of four herbs: *Scutellaria baicalensis Georgi*, *Glycyrrhiza uralensis Fisch*, *Paeonia lactiflora Pall*, and *Ziziphus jujube Mill* in the ratio of 1.5 : 1.0 : 1.0 : 1.0.

Researchers from Yale investigated if use of PHY906 could reduce the nonhaematological side effects of chemotherapy in particular GI problems. Surprisingly, in the preclinical mouse cancer model, NCr-nude mice bearing human HepG2 tumor and treated with herb-drug combinations including PHY906/CPT-11, PHY906/Capecitabine, PHY906/Doxorubicin, and PHY906/Thalidomide showed that the integrated treatment not only greatly alleviated side effects of the chemotherapy, but that PHY906 could actually potentiate tumor inhibition in a significant way. However, the nude mice used in the model have limited host cellular immunity and reduced humoral immunity, and so the model does not completely reflect human cancer patients who have intact but progressively defective immunity [24].

Encouraged by the *in vivo* findings, attempts were made to determine if the mouse model findings could be replicated in human clinical trials. Four clinical trials using different combinations of PHY906 and chemotherapeutic agents have been reported [18] showing promising results and are described in Section 2.3 and Table 2.

Other preclinical animal studies have shown that the use of PHY906 is not restricted to targeting HCC but is also active in other tumor models such as colorectal cancer and pancreatic cancer [27, 30]. In summary, although PHY906 used alone had no significant effect on tumor weight loss, it can work as an effective broad-spectrum adjuvant to enhance the chemotherapeutic efficacy of a variety of anticancer agents. In addition, other animal experiments of colorectal cancer indicated that PHY906 could reduce chemotherapy-induced toxicities by exhibiting anti-inflammatory effects and promoting the regrowth of intestinal progenitor/stem cells [23, 30] while not affecting the metabolism and antitumor activity of commonly used chemotherapeutic drugs.

#### 2.2.5. Ganfujian

Ganfujian is another CHM formula commonly used as a therapy for liver diseases. The main components are three herbs *Rhizoma Dioscoreae*, *Fructus Crataegi*, and *Fructus Ziziphi Jujubae*. These herbs are not toxic and may be used as dietotherapy for prolonged periods [31]. A preclinical study to investigate the inhibitory effect of Ganfujian granule on diethylnitrosamine- (DEN-) induced hepatoma in SD rats involved the use of 165 rats with free access to water containing 0.1 g/L DEN for 16 weeks, which were assigned into two groups to receive either normal diet or Ganfujian, respectively [19]. Thirty rats from each group were sacrificed at week 20 to observe incidence rate of liver cancer. Observation of the remaining animals continued to determine survival until week 28. At week 20, all 30 rats in the normal diet group had developed liver cancer, in comparison with 24 of 30 rats in the Ganfujian group (*P* < 0.05). The longest survival time of rats was 28 weeks in the Ganfujian group and 20 weeks in the control group (*P* < 0.05). The results suggested that Ganfujian could reduce and delay the incidence of hepatoma in rats and prolong survival time of hepatoma-bearing rats. The researchers found that Ganfujian could affect the cancer cell cycle by suppressing overexpression of related modulators such as cyclin D1 and CDK4, which is a potential antitumor mechanism [19]. Ganfujian granule is a promising liver cancer chemical preventive agent, with potential for clinical application.

### 2.3. Clinical Trials

The selected articles included clinical trials investigating 4 CHM extracts or formulas and involving a total of 216 liver cancer patients. The CHM medications tested were Huachansu injection, Kanglaite capsule, PHY906, and Jinlong capsule (Table 2).

#### 2.3.1. Huachansu

Huachansu (Cinobufacini), which is a water extract of Bufo toad skin, is used in CHM to treat conditions including swelling, pain, and heart failure [32]. Huachansu is commercially prepared for injection and is widely used at oncology clinics in China [33]. It is reported to have good effects in eliminating toxicity, as well as relieving swelling and pain. An *in vitro* pharmaceutical study of Huachansu conducted in China identified bufalin as a major anticancer component of Huachansu [34].

In a clinical trial of 11 HCC patients with stage III or IV disease who received Huachansu as a single agent, 6 patients were found to have stable disease with a response duration of 5.5 months to 11.1 months [25]. One patient whose response lasted for 11.1 months had a 20% reduction in tumor mass. Patients with stable disease had improved quality of life as assessed by using the M.D. Anderson Symptom Inventory (MDASI) scores. There were no drug-related toxicity greater than grade II and no dose-limiting toxicities reported. There was a dose-dependent increase in bufalin levels, with a maximum level of bufalin reached two hours after infusion. Further clinical studies of Huachansu with larger sample sizes and including appropriate control arms are needed. An NIH-funded project on pancreatic cancer using Huachansu has recently been completed and the report will soon be available [35]. This may shed light on the mechanism of action of clinical efficacy of Huachansu on solid tumors.

#### 2.3.2. Kanglaite

Animal experiments with Kanglaite, an acetone extract of CHM *Semen Coicis, *were described in Section 2.2 and Table 1. In a randomized controlled trial in China, 65 unresectable stage II or III HCC patients were enrolled to receive either Kanglaite with TACE (30 patients) or TACE alone (32 patients) [26]. The method of randomization was not disclosed, and it was unclear whether the trial had used a blinded method to assess the outcome. It was reported that three subjects withdrew because of financial problems. The response rates of tumors in the combination group and the TACE group were 40% and 25%, respectively (*P* > 0.05). Serum alpha fetoprotein (AFP) levels in the combination group and the TACE group were 73.1% and 60.7% lower following therapy (*P* > 0.05). Kanglaite combined with TACE had a higher median time to progression (TTP) (7.0 months) than TACE alone (5.5 months) (*P* < 0.05). Compared with TACE alone, Kanglaite plus TACE improved immune function significantly by increasing the indexes of CD3^+^, CD4^+^, and CD4^+^/CD8^+^ (*P* < 0.05). The TACE-induced adverse reaction of liver damage was less serious in the combination group than that in the TACE group. Further trials are needed with improved methodological quality so that definite conclusions on the clinical benefits of using Kanglaite in HCC can be assessed.

#### 2.3.3. PHY906

To ensure reproducible clinical efficacy, the researchers first attempted to determine the batch-to-batch consistency among different batches of PHY906 through strict quality control Good Manufacturing Practices (GMP) production measures [27]. They introduced a comprehensive technology platform termed “PhytomicsQC,” which is a multifaceted approach integrating chemical and biological fingerprinting. Together with a novel statistical analysis, the researchers showed that PhytomicsQC was useful to evaluate different batches of PHY906 and provide a robust platform to determine the batch-to-batch consistency of the four-herb CHM formula PHY906 effectively [18]. The researchers also suggested that *in vivo *animal testing can be viewed as the ultimate quality control platform, if there is a discrepancy in the chemical and biological fingerprinting results.

The clinical trials of PHY906 not only demonstrated reduced chemotherapy-induced gastrointestinal toxicity but also reported an overall stronger protective effect of global toxicity [30]. Two open-labeled clinical studies of PHY906 with capecitabine were conducted in patients with unresectable HCC [28]. The first study was a phase I/II study with 93% of patients being enrolled in the US, whereas the second study was a phase II study in Taiwan. The phase I/II trial was a multicenter, open-label, dose-escalation, safety and efficacy study of PHY906 plus capecitabine. Only 3 patients from the Taiwan site were enrolled in the phase II study, and these Taiwanese patients were excluded from the data analysis as the enrollment criteria were slightly different from those for US subjects. Of the 18 US patients enrolled in a phase I trial to determine a safe and tolerable dosing regimen, it was found that the combination of PHY906 800 mg bid and capecitabine 750 mg/m^2^ bid was well tolerated.

Subsequently, 39 patients using the recommended dose were enrolled in a phase II trial to determine whether PHY906 enhances the response rate of capecitabine, overall survival time (OS), time to disease progression (TTP), and quality of life (QoL) of patients. One of the major limitations of this trial is that there was no control arm for comparison. The disease control rate was 65.2% with 8.7% having a moderate response (MR) and 56.5% exhibiting stable disease (SD). The median TTP was 3.4 months and median OS was 9.2 months. The 12-month survival rate was 44.5%. No patients experienced drug-related grade 4 or 5 toxicities. Patients' QoL evaluated by functional assessment of cancer therapy-hepatobiliary (FACT-Hep) did not deteriorate significantly and changes in score did not exceed 25%. In subgroup analysis, the median OS of patients with hepatitis B or C was 13.8 months and nonhepatitis patients exhibited a median OS of 7.6 months. Median OS values for Child-Pugh A and Child-Pugh B patients were 10.9 and 6.5 months, respectively. The researchers stated that no formal statistical analysis was performed in this study.

The same study reported that, surprisingly, Asian patients had a higher median OS (16.5 months) than non-Asian patients (6.2 months) (*P* = 0.03). This significant survival benefit in Asian patients may be associated with their genetic phenotype of PHY906-sensitive, but further investigation with a larger patient sample is needed. The previously mentioned PHY906 clinical trials had very good quality control with consistently prepared 4-herb products, but some lacked appropriate control arms and involved relatively small numbers of patients. Therefore, future phases II and III double blind, randomized, placebo-controlled studies with sufficient patient populations are required to determine the efficacy of PHY906 in liver cancer therapy.

#### 2.3.4. Jinlong

Jinlong capsules have been commonly used as a CHM therapy for liver cancer. The major constituents are three CHM products *Gekko japonicas Dumeril et Bibron*, *Bungarus Parvus*, and *Agkistrodon*, which are all derived from reptiles. CHM theories maintain that some animal-derived medicines, especially from reptiles, can produce stronger and more deeply penetrating effects compared with those derived from plants [36]. Such CHMs are used to reduce or eliminate toxicity, activate meridians, and reduce or eliminate tumor nodes.

A randomized controlled trial (RCT) conducted in China enrolled 98 patients with liver cancer [29], of whom 53 received TACE plus Jinlong capsules and the other 45 received TACE alone. Randomization was achieved by using a table. The total response rate including both complete responses and partial responses of Jinlong in combination with TACE was 60.38% and RR of TACE alone was 40.00% (*P* > 0.05). The QoL of all patients before and after treatment was evaluated using Karnofsky (KPS). The KPS score of the combination group was higher than the TACE group after treatment (84.35 ± 12.19 versus 69.86 ± 11.58; *P* < 0.05). After treatment, the level of serum osteopontin (OPN) which relates with tumorigenesis, invasion, and metastasis was lower in the combination group than in the TACE group (117.69 ± 78.50 *μ*g/L, 151.09 ± 83.90 *μ*g/L, resp.; *P* < 0.01). The researchers did not report any adverse reactions. So, this trial suggests that short-term clinical efficacy and QoL in liver cancer can be improved by Jinlong capsules combined with TACE. Primary long-term outcomes such as overall survival time should be observed to provide further evidence of including Jinlong for liver cancer therapy.

## 3. Lessons Learned

Evidence-based clinical efficacy is the most important key criterion to be evaluated to establish whether CHM can benefit liver cancer management. Ideally the Chinese herbs chosen should comply with Chinese medicine theory in a holistic way (Section 3.1). To ensure consistent desirable clinical effects as well as safety and batch-to-batch consistency, quality control of CHM preparations is crucial (Section 3.2). As most regimens require use of CHM together with other chemotherapeutic agents, herb-drug interaction is another key factor in evidence-based CHM cancer management (Section 3.3). This section also acknowledges the multitargeted, multidimensional features of CHM by highlighting the related mechanisms of action and some possible active ingredients (Section 3.4). Other factors such as the ethical and regulatory issues of CHM products and the safe usage of health products have been addressed by other reviews [37] and will not be further discussed here.

### 3.1. Nature of the Chosen CHM

Why did the researchers choose the herbs described earlier for preclinical and clinical studies? Generally, the reason for choice of herbs originates from CHM theory. The herbs can be classified into several groups in terms of CHM thinking. *Semen Coicis*, *Rhizoma Dioscoreae*, *Glycyrrhiza uralensis Fisch, Paeonia lactiflora Pall*, and *Ziziphus jujube Mill* belong to herbs thought to strengthen healthy Qi. In contrast, *Scutellaria baicalensis Georgi*, *Bufo bufo gargarizans* Cantor, and *Scutellaria barbate D. Don* are herbs able to clear heat and remove toxins, while *Gekko japonicas Dumeril et Bibron*, *Bungarus Parvus*, and *Agkistrodon *reduce phlegm and soften hard solid mass. In the studies reviewed, herbs for strengthening healthy Qi appear to modulate immunity or affect the process of cancer cell cycle, while the heat-reducing and detoxifying herbs modulated immunity or induced cancer cell apoptosis. The anticancer mechanisms of herbs of reducing phlegm and softening hard solid mass were not reported in the animal studies. Choice of herbs used should also be in accordance with TCM Syndrome Differentiation, a theory that involves the categorization of patients into different patterns of unbalanced homeostasis, which is a vital element of TCM practice [38].

### 3.2. Quality Control of CHM to Ensure Batch-to-Batch Consistency of Clinical Efficacy

Quality control is crucial for ensuring the safety and efficacy of CHM [39]. Consistent CHM batch preparation is essential for the reliability of clinical and preclinical studies. However, many studies including some described in this review fail to mention the quality control aspects of the CHM herb of interest or the combined complex multiple-herb CHM formula. This is understandable as CHM formulas may contain hundreds of different components. In addition, the majority of active ingredients related to the effective use of CHM in disease treatment are currently unknown. Thus, to control the production of such complex matrices of diverse compounds in single herb or herbal formulae with predictable clinical efficacy is extremely difficult or even not possible.

At present, identification of the major components in the CHM preparations is by means of an array of fingerprint technologies accredited by the World Health Organization as evaluation tools [40]. These fingerprinting techniques comprise high-performance liquid chromatography, capillary electrophoresis, gas chromatography, X-ray diffraction, and DNA fingerprinting [41]. However, it should be noted that major “peaks” or “features” may not have any relationship with the bioactivities or the desirable clinical effects of that particular herb or formula.

The studies of PHY906 highlight the use of “PhytomicsQC” that integrates both chemical and biological fingerprinting to evaluate different batches of PHY906 in order to effectively provide a robust quality control platform of batch-to-batch consistency of the four-herb CHM formula [21]. Another approach advocated by Chau and his team was the use of “Quantitative-Pattern-Activity-Relationship (QPAR) [42, 43]. Using whole herbal medicines chromatographic/fingerprint profiles and their corresponding total biological activities as input, by means of sophisticated chemometrics computation, the QPAR approach can be used to explore and exploit the relationship between the CHM whole fingerprint profiles and their biological activities. Firstly, QPAR can reveal the important multiple features in the chromatographic profiles responsible for the biological activities, secondly, build a model for activities prediction simply using the chemical fingerprinting profiles, and thirdly, discover the active ingredients of the HM by identifying the multiple genuine “active” regions on the chromatogram, which are not necessarily the major components. In summary, future clinical trials using CHM should consider use of either “QPAR” or “PhytomicsQC” or similar platforms for the quality control of the batch to batch consistency to ensure reproducible clinical efficacy.

Other than quality control issues mentioned earlier, a few other important areas also require stringent control and management in order to obtain excellent batch-to-batch consistency. These areas comprise good manufacturing process conditions for high standard production and good agricultural practice to safeguard standardized plant cultivation.

### 3.3. Herb-Chemotherapeutics Interactions

Since CHMs have commonly been used in an integrative way with standard chemotherapeutics in cancer management protocols, it is pertinent to investigate if any herb-drug interaction is occurring. From both the physicians' and patients' points of view, it is important to understand if the CHMs affect the pharmacokinetics of the chemotherapeutic agent by reducing the level of chemotherapeutics in the patients' blood leading to failure to provide enough dosage inside the cancer cells. Conversely, herb-drug interaction may also potentially relate to the drug safety, if the herb unexpectedly enhances absorption of the chemotherapeutic agent. For instance, in a clinical trial of lung cancer, an astragalus-based herbal formula, Jinfukang, was shown to alter the pharmacokinetics of docetaxel, with most patients experiencing increase in docetaxel levels by at least 33%, although no clear trend was evident [44].

Currently, there are few reports describing this key aspect of herb-drug interaction. One study has addressed the effects of PHY906 on the pharmacokinetics of CTP-11, capecitabine, gemcitabine, and sorafenib in colorectal cancer, HCC, and pancreatic cancer, respectively, in animal models [21]. Importantly, results indicated that PHY906 did not affect the metabolism of these chemotherapeutic agents or their corresponding metabolites. However, it has to be noted that these findings were based on animal studies only, and it is therefore important that a similar approach should be adopted for human clinical trials. There is a clear need for more systematic coordinated herb-chemotherapeutic interaction studies to provide an in-depth knowledge base about use of combination therapy.

### 3.4. Mechanistic Evaluation of CHMs Leading to Personalized Integrative Medicine

Traditional Chinese Medicine theory proposes CHM formulae may provide holistic, multitargeted, multidimensional pharmacological therapies leading to effective cancer management. Accordingly, the mechanisms of action of CHM for liver cancer are also likely to be multifactorial. As shown in Tables 1 and 2, the potential mechanisms of action include anti-inflammation, antiangiogenesis, antivirus, apoptosis-induction, cell cycle arrest, modification of tumor microenvironment, and immune-modulation.

To date, relatively few studies have examined the mechanistic effects of CHM on the immune response, and the specific target proteins or the underlying related signal transduction pathways affected by CHM have also not been addressed. This may be due to the current poor understanding of the actual targets of the multiple active compounds of the CHM responsible for the clinical effects. A coordinated network of laboratories collaborating to determine these effects is urgently needed.

Methodologies to approach these multitargeted, multidimensional pharmacological activities of CHM have been proposed by several research groups [45–47]. These platforms make use of recent advances in “Omics” bioinformatics, pharmacogenomics, and systems biology, which have all been found to be useful for the analysis of diverse complex data [48].

Furthermore, as CHM targets the underlying disturbed homeostasis, studies concerning the mechanisms related to the characteristics of TCM Syndrome Differentiation that reflect the inner health status of the body should be conducted [49]. Recent reports discovering “phenotypes” based on the systems biology approach may be the beginning of a new frontier of scientific research into this important aspect of TCM Syndrome Differentiation [50, 51]. A sophisticated and fuller understanding of Syndrome Differentiation may lead to TCM-based personalized treatment strategies for HCC CHM or integrative treatment, or it may provide the rationale for patient stratification of groupings with similar homeostasis imbalances for interpretation of clinical trials.

## 4. Discussion

This review focused on some CHM therapies reported to have significant effects for liver cancer. These therapies have not yet gained wide acceptance as part of integrative liver cancer management. The explanation for this is multifaceted.

First, many publications of the use of CHMs in liver cancer management are written in Chinese; of the 139 articles excluded from this review, 85 were rejected due to this language criterion. These papers could potentially include useful information; however, they may suffer from the severe inadequacy of lack of independent assessment for veracity and reproducibility by western scientists [52].

Second, this review highlights preclinical studies using various laboratory experimental platforms and animal cancer studies, which are important for providing evidence of the efficacy and mode of action of CHMs in liver cancer clinical trials. This approach illustrates the potential of reverse-translational research. Laboratory experimental platforms are used to study the mechanisms of action and the potential “active ingredients” in the CHMs in relation to the effective clinical trials results. Such knowledge can lead to modifications of treatment and assist in the quality control of the CHM using defined amounts of those “active ingredients.” This knowledge can then be applied to the patients, leading to improvement and consistency of both clinical efficacy and safety.

Third, although some of the clinical studies included in this review suffered from problems of study design or other quality issues, they nevertheless provide credible evidence suggesting effectiveness of some selected CHMs. Further trials ensuring high quality control of production of CHM agents to reduce the batch-to-batch variations and rigorously designed randomized, controlled, multicentre trials are required.

In this review, most clinical studies examined the effectiveness of CHM therapies for liver cancer. In TCM, the medicines are prescribed according to syndrome. Syndrome differentiation remains the essence of CHM treatment and is the key to evaluating a patient's disease state and developing an efficient, individualized treatment strategy. Therefore, syndrome differentiation should be applied to the treatment of liver cancer by CHM, possibly using a personalized treatment plan or a set of plans for a few defined syndrome profiles. We believe that the application of TCM syndrome differentiation to disease diagnosis can yield improved therapeutic efficacy. We also envision that TCM syndrome will be able to be assessed by modern technologies in the future. The diagnosis and efficacy of CHM therapies for liver cancer can then be evaluated on a molecular and Systems biology basis.

## 5. Conclusions

In this study, we systematically reviewed some CHMs for the treatment of liver cancer. We first summarized the CHM extracts or formulas in preclinical studies and clinical trials and evaluated these studies with focus on the mechanisms of action and TCM theory. Analysis of these clinical trials revealed the need for cautious interpretation of results as apparent benefits may actually attributable to various forms of bias inherent in the study designs. Overall these CHM agents appeared to be potentially beneficial, and further well-designed and controlled randomized clinical trials should be performed to enhance the creditability of CHM treatment. This critical analysis of the CHM anti-HCC research covered methodology in clinical trials, TCM Syndrome Differentiation and prescription, quality control for therapeutic consistency, assessment of herb-drug interactions, and the outlined shortcomings. The learned lessons based on the suggested improvements may contribute to the enhancement of developing a comprehensive evidence-based liver cancer CHM research platform.

## Figures and Tables

**Table 1:** Preclinical studies of CHM in liver cancer.

Name	Composition	Biological action	Preclinical study
Bufalin [14]	*Secretia bufonis *	Anticancer	Inhibited growth of tumor in *in situ* transplantation tumor model of nude mice Prolonged survival time of hepatoma bearing mice Induced apoptosis through regulating expression of apoptosis-related gene *bax* and the ratio of bcl-2/bax *in situ* hepatoma transplantation model and apoptotic characteristics determined by ultrastructure observation

Kanglaite injection [15, 16]	Semen Coicis	Anticancer	Prohibited growth of transplant hepatoma in rats by intra-tumor injection Inhibited expression of PCNA of transplanted hepatoma in rats Reduced liver toxicity compared to ethanol therapy Induced apoptosis in cancer cell through activation of the Fas/FasL pathway

Banzhilian [17]	*Scutellaria barbate D. Don *	Anticancer, Immune modulation	Inhibited growth of tumors in hepatoma bearing mice Improved phagocytic function of macrophages determined by chicken-red cell phagocytic rate Inhibited proliferation of cancer cells *in vitro* Induced apoptosis determined by ultrastructure observation of apoptotic cancer cells

PHY906 [18]	*Scutellaria baicalensis Georgi, Glycyrrhiza uralensis Fisch, Paeonia lactiflora Pall, Ziziphus jujube Mill *	Anticancer	Inhibiting growth of tumors in mice bearing human HepG2 tumor and enhancement of chemotherapeutic efficacy when combined with anticancer agents such as CPT-11, capecitabine, doxorubicin, and thalidomide

Ganfujian granule [19]	*Rhizoma Dioscoreae, Fructus Crataegi, Fructus Ziziphi Jujubae, etc. *	Anticancer	Reduced and delayed the incidence of hepatocellular carcinoma in rats Prolonged survival time of tumor bearing rats Affected process of cancer cell cycle through suppressing overexpression of related modulators

**Table 2:** Clinical studies of CHM in liver cancer.

Name	Composition	Biological action	Clinical study
Huachansu injection [25]	*Bufo bufo gargarizansCantor *	Anticancer Anti-HBV	11 patients with stage III or IV 6 had response of prolonged SD SD time is 5.5 to 11.1 months 1 had a 20% regression of tumor Patents with SD had improved quality of life No dose-limiting toxicities found No drug-related toxicity greater than grade II

Kanglaite capsule [26]	*Semen Coicis *	Anticancer Immune modulation	65 patients with stage II or III Tumor RR for combination and TACE alone were 40% and 25% AFP RR for combination and TACE alone were 73.1% and 0.7% TTP for combination and TACE alone were 7.0 and 5.5 months Improved immune function evaluated by CD3^+^, CD4^+^, and CD4^+^/CD8^+^ Improved QoL Reduced TACE-induced adverse reaction of liver damage

PHY906 [27, 28]	*Scutellaria baicalensis Georgi, Glycyrrhiza uralensis Fisch, Paeonia lactiflora Pall, Ziziphus jujube Mill *	Anticancer, Anti-inflammatory Immune modulation	18 advanced liver cancer patients were enrolled in phase I study to determine a safe and tolerable dose 39 advanced liver cancer patients were enrolled in phase II study to observe tumor response, OS, TTP, and QoL No patient experienced drug-related grade 4 or 5 toxicities Disease control rate was 65.2% : 8.7% MR and 56.5% SD OS was 9.2 months TTP was 3.4 months The 12-month survival rate was 44.5% OS for HBV/HCV and non-HBV/HCV subgroups were 13.8 and 7.6 months OS for Child-Pugh A and Child-Pugh B patients were 10.9 and 6.5 months OS for Asian and non-Asian subgroups were 16.5 and 6.2 months Patients' QoL did not deteriorate significantly and changes in score did not exceed 25%

Jinlong capsule [29]	*Gekko japonicas Dumeril et Bibron, Bungarus Parvus, Agkistrodon, etc. *	Anticancer	98 liver cancer patients RR (CR + PR) was 60.38% when combined with TACE, while TACE alone was 40.00% KPS scores were higher than TACE alone No adverse reaction Jinlong capsule found Level of serum OPN was lower than that of TACE alone

TACE: transcatheter arterial chemoembolization; OS: overall survival; TTP: time to disease progression; QoL: quality of life; SD: stable disease; CR: complete response; PR: partial response; RR = CR + PR: total response rate.
